# Deletion of *Slc26a1* and *Slc26a7* Delays Enamel Mineralization in Mice

**DOI:** 10.3389/fphys.2017.00307

**Published:** 2017-05-16

**Authors:** Kaifeng Yin, Jing Guo, Wenting Lin, Sarah Y. T. Robertson, Manoocher Soleimani, Michael L. Paine

**Affiliations:** ^1^Center for Craniofacial Molecular Biology, Herman Ostrow School of Dentistry of University of Southern CaliforniaLos Angeles, CA, USA; ^2^Department of Orthodontics, Herman Ostrow School of Dentistry of University of Southern CaliforniaLos Angeles, CA, USA; ^3^Department of Endodontics, Herman Ostrow School of Dentistry of University of Southern CaliforniaLos Angeles, CA, USA; ^4^Department of Medicine, University of Cincinnati, Research Services, Veterans Affairs Medical CenterCincinnati, OH, USA

**Keywords:** amelogenesis, enamel maturation, pH regulation, bicarbonate transport, SLC26a1, SLC26A7

## Abstract

Amelogenesis features two major developmental stages—secretory and maturation. During maturation stage, hydroxyapatite deposition and matrix turnover require delicate pH regulatory mechanisms mediated by multiple ion transporters. Several members of the Slc26 gene family (*Slc26a1, Slc26a3, Slc26a4, Slc26a6, and Slc26a7)*, which exhibit bicarbonate transport activities, have been suggested by previous studies to be involved in maturation-stage amelogenesis, especially the key process of pH regulation. However, details regarding the functional role of these genes in enamel formation are yet to be clarified, as none of the separate mutant animal lines demonstrates any discernible enamel defects. Continuing with our previous investigation of *Slc26a1*^−/−^ and *Slc26a7*^−/−^ animal models, we generated a double-mutant animal line with the absence of both *Slc26a1* and *Slc26a7*. We showed in the present study that the double-mutant enamel density was significantly lower in the regions that represent late maturation-, maturation- and secretory-stage enamel development in wild-type mandibular incisors. However, the “maturation” and “secretory” enamel microstructures in double-mutant animals resembled those observed in wild-type secretory and/or pre-secretory stages. Elemental composition analysis revealed a lack of mineral deposition and an accumulation of carbon and chloride in double-mutant enamel. Deletion of *Slc26a1* and *Slc26a7* did not affect the stage-specific morphology of the enamel organ. Finally, compensatory expression of pH regulator genes and ion transporters was detected in maturation-stage enamel organs of double-mutant animals when compared to wild-type. Combined with the findings from our previous study, these data indicate the involvement of SLC26A1and SLC26A7 as key ion transporters in the pH regulatory network during enamel maturation.

## Introduction

Acid-base balance is one of the major essential processes during amelogenesis (Simmer and Fincham, 1995; Smith et al., 1996; Smith and Nanci, 1996; Lacruz et al., 2010a, 2012b), and it has been suggested that fluctuations in extracellular pH level during maturation-stage enamel development are essential for mineral growth (Simmer and Fincham, 1995). Digestion of enamel matrix proteins (EMPs) through the endosome/lysosome pathway, following trafficking from the enamel space, relies highly on the acidic intracellular luminal environment (Lloyd, 1996). Previous studies have identified the functional role of several groups of genes, including carbonic anhydrases (Lezot et al., 2008), cystic fibrosis transmembrane conductance regulator (CFTR), chloride channels (CLCNs), solute carrier gene family 4 (SLC4s) and solute carrier gene family 9 (SLC9s), in maintaining the ameloblast-mediated pH homeostasis within both extracellular space and intracellular lumens (Dogterom and Bronckers, 1983; Lin et al., 1994; Wright et al., 1996a,b; Arquitt et al., 2002; Lyaruu et al., 2008; Paine et al., 2008; Bronckers et al., 2009, 2010; Josephsen et al., 2010; Wang et al., 2010; Lacruz et al., 2010a,b, 2012c, 2013a; Chang et al., 2011; Duan et al., 2011; Duan, 2014; Jalali et al., 2014; Reibring et al., 2014; Wen et al., 2014).

The solute carrier (SLC) 26A gene family encodes multiple anion transporters with chloride/bicarbonate exchanger activities (Xie et al., 2002; Petrovic et al., 2003a,b, 2004; Alper and Sharma, 2013). Animal models with mutations of *Slc26a1, Slc26a6*, and *Slc26a7* exhibit disorders featuring disruption of ion homeostasis, such as urolithiasis, hepatotoxicity, renal tubular acidosis and impaired gastric secretion (Freel et al., 2006; Jiang et al., 2006; Xu et al., 2009; Dawson et al., 2010). Based on our previous study and those of Bronckers et al., Slc26a1/Sat1, Slc26a3/Dra, Slc26a4/pendrin, Slc26a6/Pat1 and Slc26a7/Sut1 are immunolocalized in secretory- and maturation-stage ameloblasts (Bronckers et al., 2011; Jalali et al., 2015; Yin et al., 2015). In particular, these genes mainly localize to the apical membrane/subapical vesicles of maturation ameloblast. In addition, the expression of Slc26a1, Slc26a6, and Slc26a7 is significantly upregulated at both RNA and protein levels during maturation stage compared to secretory stage (Yin et al., 2014, 2015). These are strong indications of the functional involvement of the Slc26 gene family in pH regulation during amelogenesis. However, the deletion of these genes individually fails to induce any abnormal enamel phenotypes, likely due to the compensatory expression of other pH regulatory genes and Slc26a isoforms, suggesting a yet-to-be-identified master pH response regulatory mechanism in amelogenesis (Bronckers et al., 2011; Jalali et al., 2015; Yin et al., 2015).

In this study, we generated an animal model with the absence of both *Slc26a1* and *Slc26a7* by breeding homozygous parents (*Slc26a1*^−/−^ and *Slc26a7*^−/−^). We showed that the double-null enamel density was significantly lower in the regions that represent late maturation-, maturation-, and secretory-stage enamel development in wild-type mandibular incisors. However, the “maturation” and “secretory” enamel microstructures in double-mutant animals resembled those observed in wild-type secretory and/or pre-secretory stages. Elemental composition analysis revealed a lack of mineral deposition and an accumulation of carbon and chloride in double-mutant enamel, although absence of *Slc26a1* and *Slc26a7* did not affect the stage-specific morphology of the enamel organ including ameloblasts. Finally, compensatory expression of pH regulators and ion transporters at RNA level was detected in maturation-stage enamel organs of double-mutant animals. Taken together, the data obtained from double mutant animals (*Slc26a1*^−/−^ and *Slc26a7*^−/−^) provide new evidence from a functional perspective to support the hypothesis that SLC26A1/SAT1 and SLC26A7/SUT1 are actively involved in ameloblast-mediated pH regulation during maturation-stage amelogenesis.

## Materials and methods

### Animals

All vertebrate animal manipulation was carried out in accordance with Institutional and Federal guidelines. The animal protocols were approved by the Institutional Animal Care and Use Committee at the University of Southern California (Protocol # 11736). For immunofluorescence analysis, we dissected mandibles and kidneys from rats (Wistar Hannover, 4-week, 100–110 g). *Slc26a1*^+/−^ mice were purchased from the Jackson Laboratory (stock # 012892) and *Slc26a7*^+/−^ mice were a kind gift from Dr. Manoocher Soleimani (Xu et al., 2009; Dawson et al., 2010). *Slc26a1*^−/−^ and *Slc26a7*^−/−^ mice were generated by breeding heterozygous (*Slc26a1*^+/−^ or *Slc26a7*^+/−^) parents. To generate double-mutant animals with the absence of *Slc26a1* and *Slc26a7*, we crossed *Slc26a1*^−/−^ and *Slc26a7*^−/−^ mice. The double-mutant lines were genotyped by PCR using primers designed in earlier studies (Xu et al., 2009; Dawson et al., 2010).

### Immunofluorescence

The expression patterns of Slc26a1 and Slc26a7 in maturation-stage ameloblasts were shown by co-localization using immunofluorescence (IF). Hemi-mandibles and kidneys obtained from Wistar Hannover rats (100–110 g body weight, 4 weeks old) were fixed in 4% paraformaldehyde (PFA) at 4°C overnight. The hemi-mandibles were then decalcified in 10% EDTA (pH 7.4) for 2 months. Sagittal sections were prepared from paraffin-embedded tissue blocks with a thickness of 7 μm. After being dewaxed, rehydrated and blocked by 1% bovine serum albumin (BSA) in PBST (1X, pH 7.4), the tissue sections were incubated with primary antibodies against Slc26a1 (Santa Cruz Biotechnology, Catalog # sc-132090, dilution 1:400) and Slc26a7 (Abcam, Catalog # ab65367, dilution 1:300). All tissue sections were stained with DAPI (Vector Laboratories; Catalog # H-1200) before cover slides were applied.

### μCT analysis

Mandibles were dissected from 4-week-old double-mutant animals and their age-matched wild-type controls. Samples from 12 animals in each group were prepared for μCT analysis (*n* = 12, SkyScan 1174) with the scanner setting to 50 kVp, 800 μA, and 6.7 μm resolution. The reconstruction and calculation of the enamel density of mandibular incisors and first molars were performed with Amira 3D Visualization and Analysis Software 5.4.3 (FEI Visualization Science Group, Burlington, MA, USA) (Wen et al., 2015). The potential statistical differences in the relative enamel density between double-mutant and wild-type groups were evaluated by a two-tailed Student's *t*-test using IBM SPSS Statistics 22.0 (significance level defined as *P* < 0.05).

### Scanning electron microscopy and energy-dispersive X-ray spectroscopy (EDS)

The hemi-mandibles prepared for μCT analysis (*n* = 12) were used for the subsequent SEM and EDS analyses. The samples were scanned and imaged by SEM and EDS according to previously published protocols (Lacruz et al., 2010b; Wen et al., 2014; Yin et al., 2015).

### Hematoxylin and eosin (H & E) staining

Mandibles were dissected from 4-week-old double-mutant animals and wild-type controls for H & E staining. The protocols followed those described in a previous study (Lacruz et al., 2012a).

### Realtime PCR analysis

RNA samples of maturation-stage enamel organs were extracted from mandibles of double-mutant and wild-type animals (*n* = 6) using a method described previously (Yin et al., 2015). cDNA used for real-time PCR analysis was prepared using the miScript II RT Kit with miScript HiFlex Buffer (Qiagen). To detect the expression changes in the genes that have been identified to be involved in maturation-stage pH regulation (Dogterom and Bronckers, 1983; Wright et al., 1996a,b; Andrejewski et al., 1999; Arquitt et al., 2002; Lyaruu et al., 2008; Paine et al., 2008; Bertrand et al., 2009; Bronckers et al., 2009, 2010, 2011; Josephsen et al., 2010; Wang et al., 2010; Lacruz et al., 2010a,b, 2011, 2012b,c, 2013b; Chang et al., 2011; Duan, 2014; Jalali et al., 2014; Yin et al., 2014, 2015), real-time PCR reactions were performed on a CFX96 TouchTM Real-Time PCR Detection System (Bio-rad Life Sciences) with iQ SYBR® Green supermix (Bio-rad Life Science) and mouse-specific primers (Table 1). The Ct values were normalized to those of *Actb* (*Beta-actin*). The ΔΔCt method was used to calculate the fold changes in gene expression (double-mutant relative to wild-type; Livak and Schmittgen, 2001; Schmittgen and Livak, 2008). Two-tailed Student's *t*-tests were used to detect the potential differences in the expression levels of gene transcripts between double-mutant and wild-type groups (significance level defined as *P* < 0.05). Data were analyzed using IBM SPSS Statistics 22.0 software.

**Table 1:** **Mouse-specific primers for qPCR**.

**Symbol**	**Accession**	**Region**	**Forward (5′–3′)**	**Reverse (5′–3′)**
*Car2*	NM_009801	4–173	TCCCACCACTGGGGATACAG	CTCTTGGACGCAGCTTTATCATA
*Car6*	NM_009802	61–160	TGGAGCTATTCAGGGGATGATG	CCGTCTTCACGTCGATGGG
*Cftr*	NM_021050	957–1,132	GCATATTGTTGGGAATCAGC	ACGATTCCGTTGATGACTGT
*Ae2*	NM_009207	3,282–3,523	CATGGAGACACAGATCACCA	GCTGTTCCTTGACTTCCTGA
*Ae4*	NM_172830	10-127	CCAGGGCAGGGGGATTTTG	CCCCAATGTCTATGCCTGAGG
*NBCe1*	NM_018760	246–489	CTCCGAGAACTACTCCGACA	ACCCTGCTCCACTTTCTCTT
*Slc26a6*	NM_134420	2,023–2,184	TTGCTGGAGCTGTATCTTCC	TGTTTGCCTTCCAAAGAGAG
*Lamp1*	NM_010684	930–1,076	TCTATGGCACTGCAACTGAA	GGCTCTGTTCTTGTTCTCCA
*Lamp2*	NM_001017959	785–916	AACTTCAACACCCACTCCAA	AAAGGCACCTTCTCCTCAGT
*Lamp3*	NM_007653	598–809	CACAGACTGGGAAAACATCC	TAATTCCCAAGACCTCCACA
*Lamp4*	NM_009853	630–811	ACATCAGAGCCCGAGTACAG	GGTGAACAGCTGGAGAAAGA
*Clcn7*	NM_011930	316–477	CCAAGGAGATTCCACACAAC	CAATGAGGGCACAGATAACC
*Rab21*	NM_024454	723–963	TCCGCTAAACAGAACAAAGG	GGCAATGATCCACAGTTCTC
*Alpl*	NM_007431	2,228–2,472	TCTGCTCAGGATGAGACTCC	TCCCTTTTAACCAACACCAA
*Nhe1*	NM_016981.2	780–972	CATCCTTGTCTTCGGGGAGTC	GGAGGTGAAAGCTGCGATTAC
*Actb*	NM_007393	792–951	AAGAGCTATGAGCTGCCTGA	TACGGATGTCAACGTCACAC

## Results

### SLC26A1 and SLC26A7 do not colocalize in maturation-stage ameloblasts

**Figure 1:**
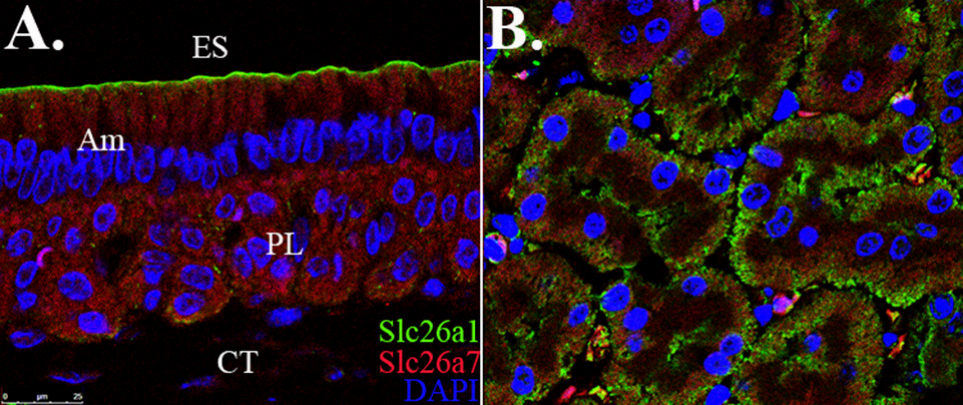
*****Colocalization analysis of Slc26a1 with Slc26a7*****. ES, Enamel space; Am, Ameloblast; PL, Papillary layer; CT, Connective tissue. **(A)** Colocalization of SLC26A1and SLC26A7 in maturation-stage ameloblasts by confocal microscopy at 63x magnification. SLC26A1 immunolocalized to the apical membrane of maturation-stage ameloblasts. SLC26A7 showed more expression in the cytoplasmic area in addition to an apical/subapical distribution. Apparent overlaps in fluorescence from SLC26A1 and SLC26A7 were not observed. **(B)** Tissue sections prepared from rat kidneys were stained with the same antibodies as a reference. All sections were counterstained with DAPI to highlight the nuclei (blue).

We revisited the expression patterns of SLC26A1 and SLC26A7 in rodent maturation-stage ameloblasts by conducting colocalization analysis using immunofluorescence. The expression of Slc26a1 was mainly immunolocalized to the apical membrane of maturation ameloblast (Figure 1A). In contrast, SLC26A7 showed more expression in the cytoplasmic area in addition to an apical/subapical distribution (Figure 1A). No apparent overlaps in fluorescence signals from SLC26A1 and SLC26A7 were observed (Figure 1A). Tissue sections prepared from rat kidneys were stained with the same antibodies as a reference (Figure 1B).

### Double-mutant mandibular incisors demonstrate decreased enamel density

**Figure 2:**
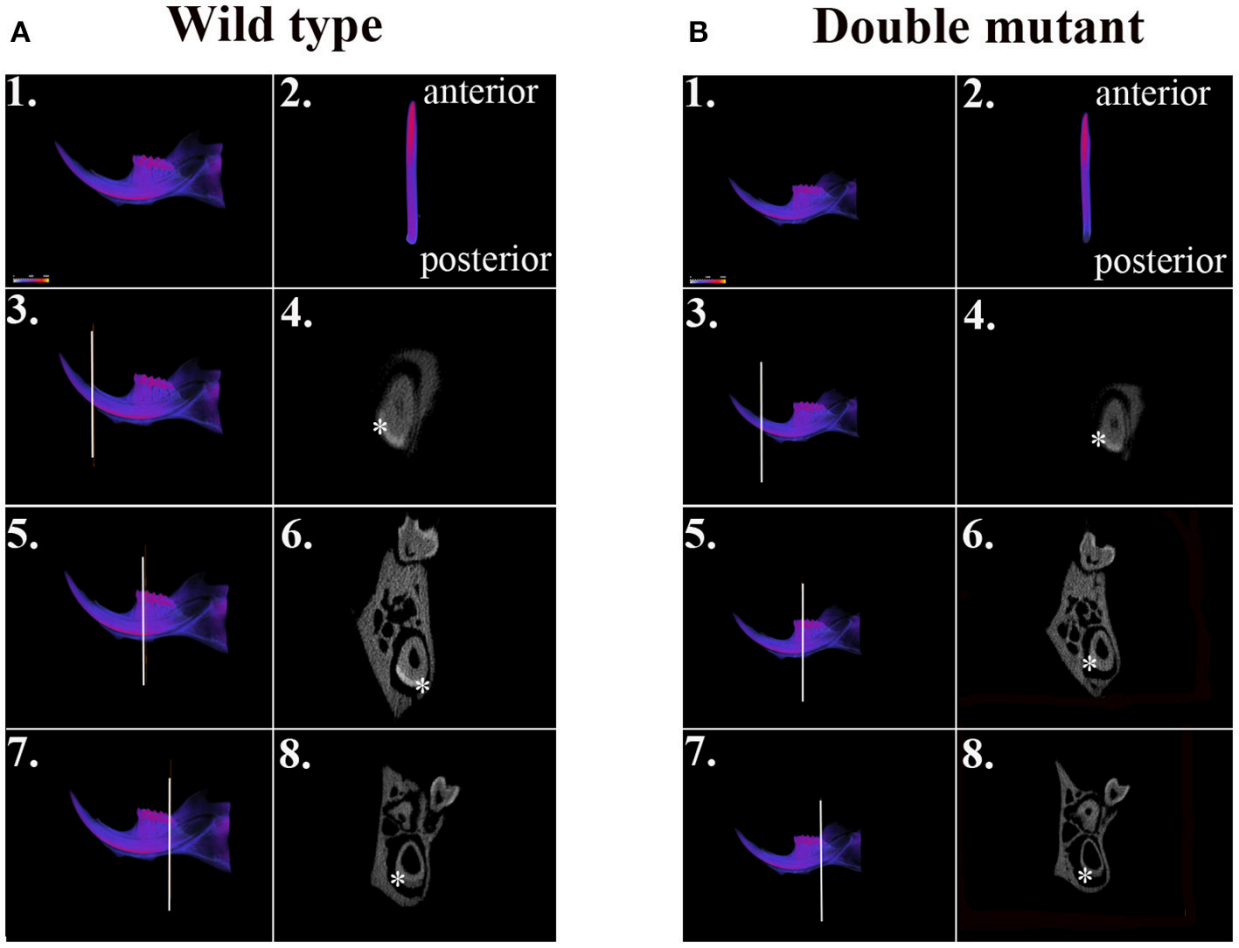
**μCT analysis of wild-type and double-mutant mandibular incisors. (A1)** Wild-type semi-mandible. **(A2)** Wild-type incisor viewed from the labial surface. **(B1)** Double-mutant semi-mandible. **(B2)** Double-mutant incisor viewed from the labial surface. We selected three regions to analyze the enamel density of incisors, which are indicated by the three reference planes along the long axis of mandibular incisors. The first reference plane was placed at the region where bony support begins **(A3,B3)**. The second and the third reference planes sectioned though the first **(A5,B5)** and the third mandibular molars **(A7,B7)**. In the wild-type sample, the three reference planes from anterior to posterior represent late maturation, maturation and secretory stages, respectively. **(A4,A6,A8,B4,B6,B8)** Are cross-sectional views of the regions of the three reference planes. Colors varying from blue to red in **(A1–A3,A5,A7,B1–B3,B5,B7)** indicate an increase in density. The area of mandibular incisor enamel is labeled by ^*^ in **(A4,A6,A8,B4,B6,B8)**.

**Figure 3:**
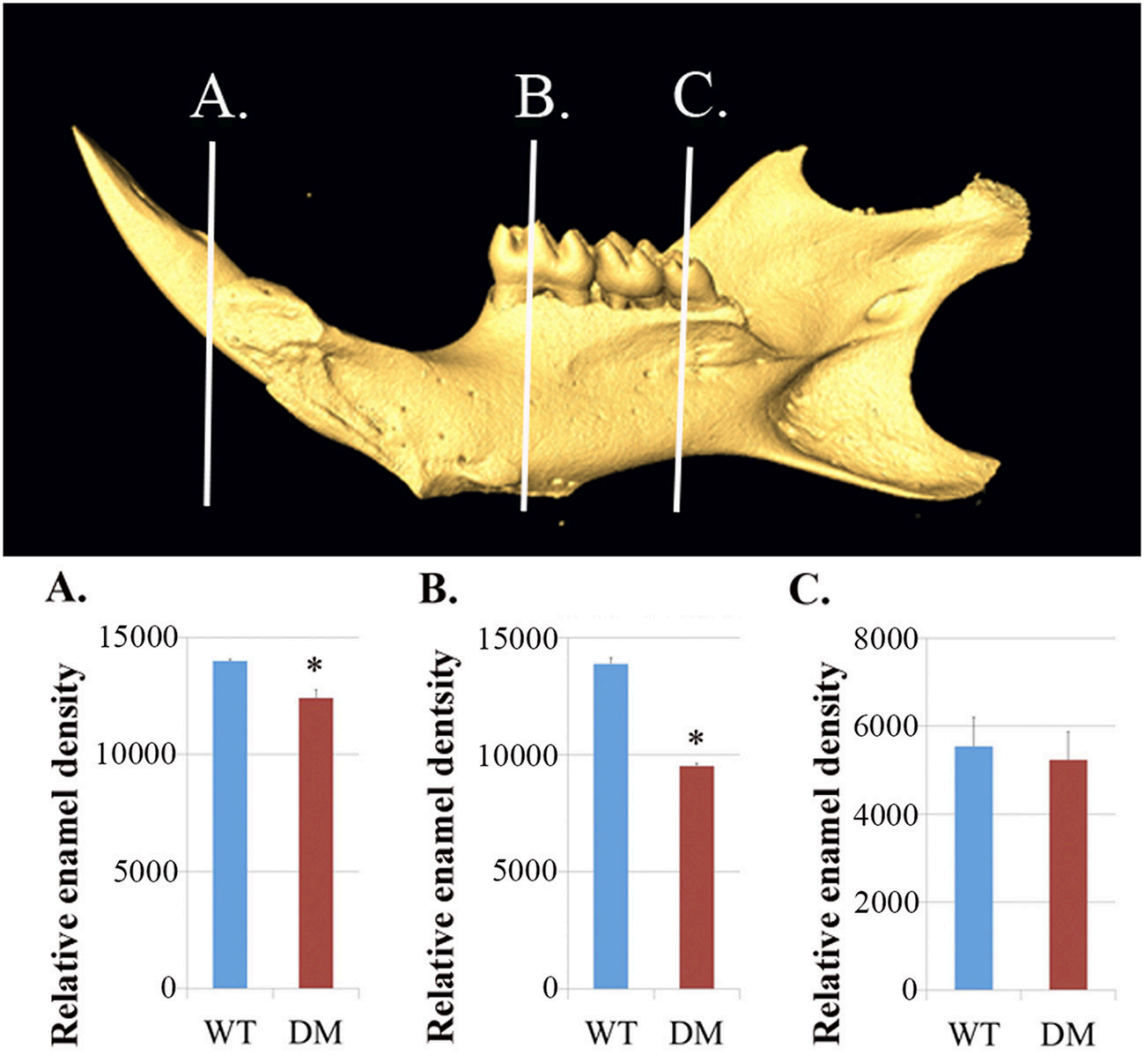
**Quantification of enamel density in wild-type and double-mutant mandibular incisors**. WT, Wild-type; DM, Double-mutant. **(A)** At the first reference plane, the double-mutant enamel density was approximately 14.3% lower than that of wild-type enamel (*P* = 0.015). **(B)** At the second reference plane, the density gap between double-mutant and wild-type enamel was even higher—35.7% (*P* = 0.010). **(C)** The difference in relative enamel density of mandibular incisors was not significant between the wild-type and double-mutant groups (*P* = 0.56). ^*^P < 0.05.

We dissected hemi-mandibles from 4-week-old double-mutant animals and their age-matched wild-type controls for μCT analysis. After 3D reconstruction from raw dicom files, we selected three regions to analyze the enamel density of incisors, which were indicated by the three reference planes along the long axis of the mandibular incisors (Figure 2). The first reference plane was placed at the region where bony support ends (Figures 2A3,B3). The second and the third reference planes sectioned though the first and the third mandibular molars (Figures 2A5,A7,B5,B7). The three reference planes from anterior to posterior represent late-maturation, maturation and secretory stages, respectively (Nanci, 2008; Lacruz et al., 2011, 2012b; Yin et al., 2014, 2015). In a 4-week-old wild-type mouse, the enamel of the mandibular incisor is fully mature (maturation-stage) between the first and the second reference planes (Figures 2A2–A6) (Yin et al., 2015). In contrast, the double-mutant enamel at the first and second reference planes showed statistically significant decreases in relative density (Figures 2B2–B6, 3A,B, *P* < 0.05). The double-mutant enamel density was approximately 14.3% lower than wild-type enamel density at the first reference plane (Figure 3A). At the second reference plane, the density gap between double-mutant and wild-type enamel was even higher—35.7% (Figure 3B). The enamel on mandibular incisors at the third reference plane is in secretory stage in wild-type animals (Yin et al., 2015), and the difference in relative enamel density of incisors was not statistically significant between wild-type and double-mutant groups (Figures 2A8,B8, 3C).

**Figure 4:**
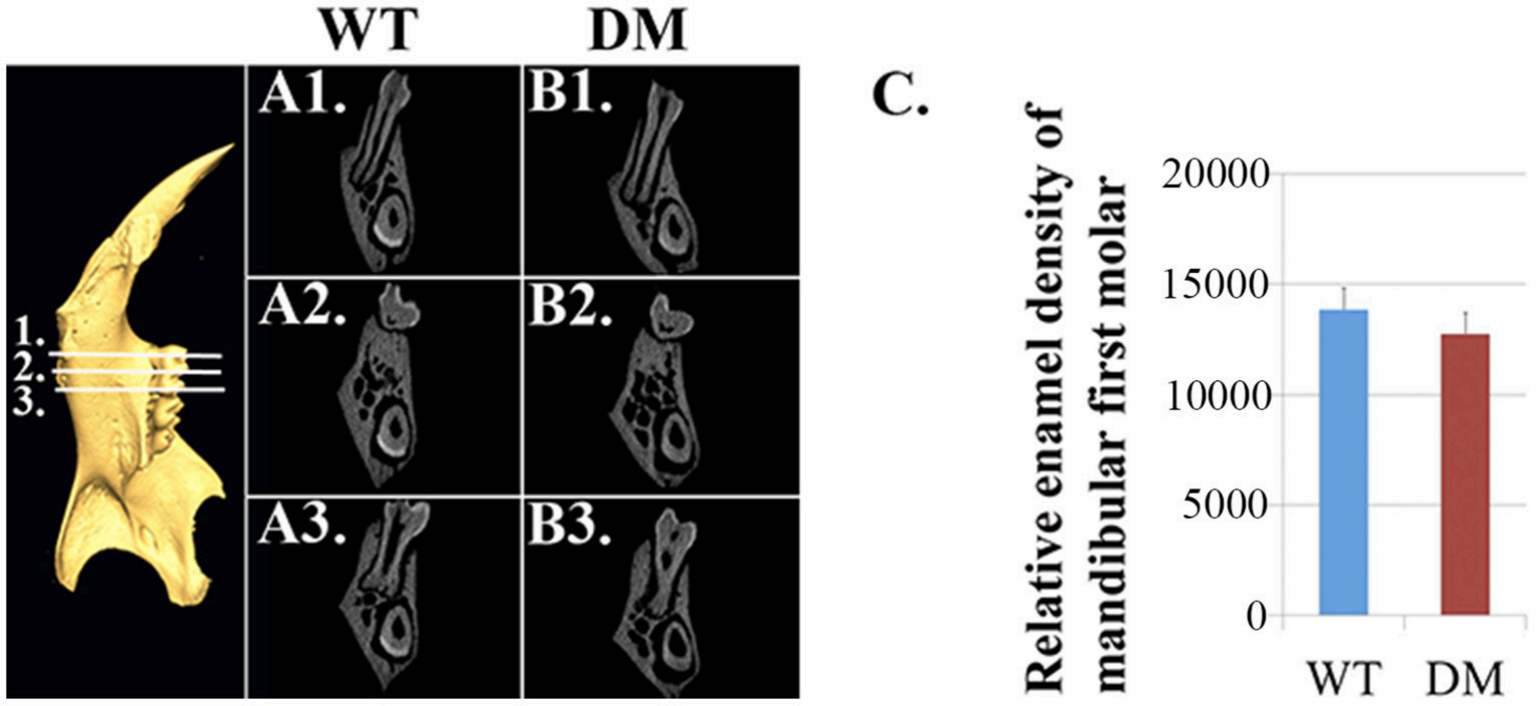
**Quantification of enamel density in wild-type and double-mutant mandibular first molars**. WT, Wild-type; DM, Double-mutant. **(A1–A3)** Cross-sectional views of wild-type hemi-mandible at reference planes 1, 2 and 3. **(B1–B3)** Cross-sectional views of double-mutant hemi-mandible at reference planes 1, 2, and 3. **(C)** We averaged the measurements obtained from mesial, middle and distal cusps (reference planes 1, 2, and 3). The double-mutant molars demonstrated lower enamel density than wild-type molars, but the difference was not statistically significant (*P* = 0.35).

Based on μCT analysis, we also quantified the relative density of mandibular first molars. For calculating the enamel density of each molar, we averaged the measurements obtained from mesial, middle and distal cusps (Figure 4). Although the double-mutant molars demonstrated lower enamel density than wild-type molars, the differences were not statistically significant (Figure 4C, *P* = 0.35).

### Absence of *Slc26a1* and *Slc26a7* disrupts development of enamel microstructure

**Figure 5:**
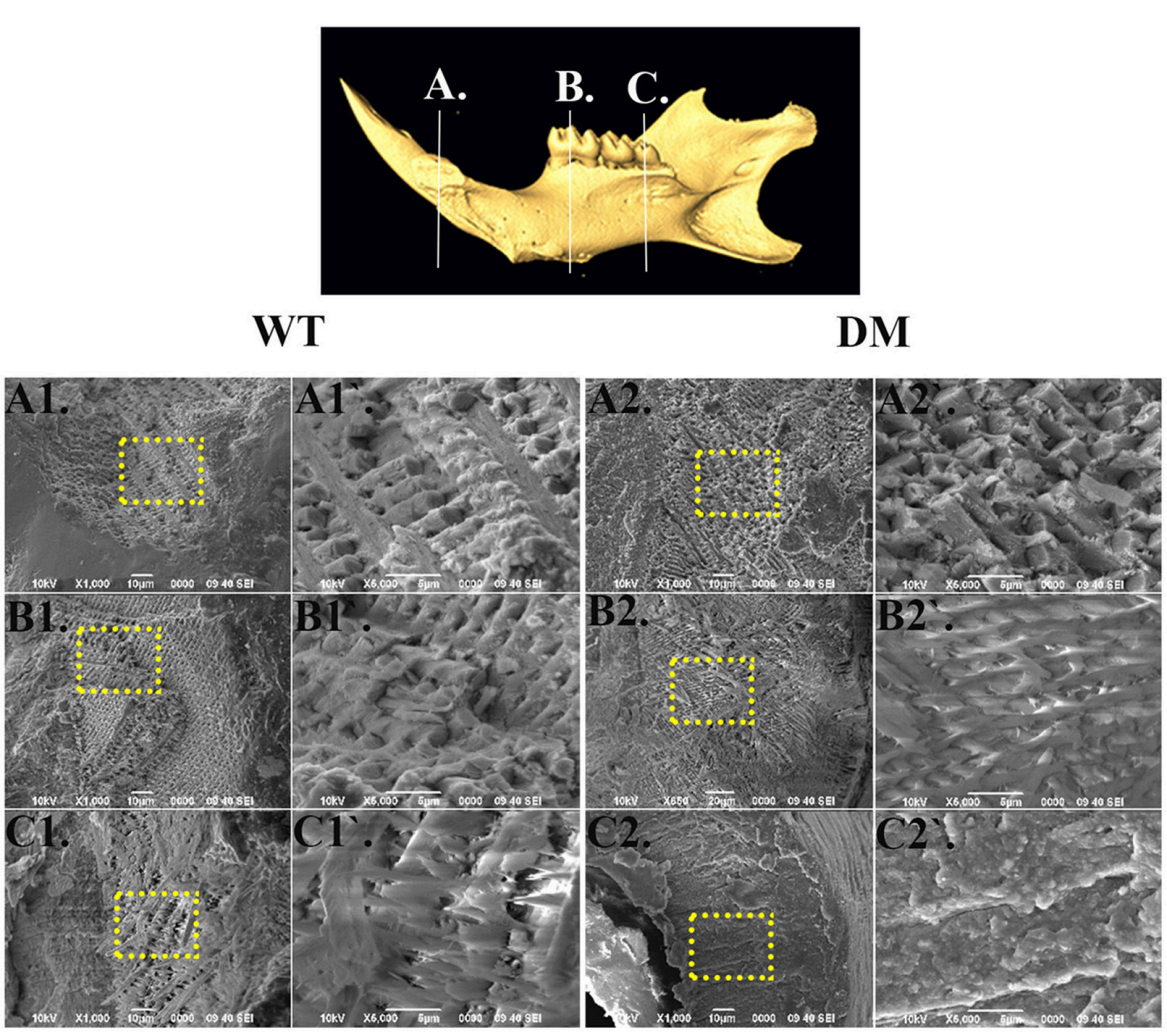
**SEM analysis of enamel microstructures in wild-type and double-mutant mandibular incisors**. WT, Wild-type; DT, Double-mutant. **(A1–C1)** Wild-type enamel at reference planes A, B, and C under 1000x magnification. **(A1'–C1')** The areas in **(A1–B1)** labeled with dotted frames under 5000x magnification. (**A2–C2)** Double-mutant enamel at the reference planes A, B, and C under 1000x magnification. **(A2'–C2')** The areas in **(A2–B2)** labeled with dotted frames under 5000x magnification. At the first and the second reference planes, wild-type enamel showed typical microstructure of maturation-stage enamel with rods and interrods laid out in a decussating and orderly pattern **(A1,A1',B1,B1)**. In wild-type enamel at secretory stage, marked by the third reference plane, enamel rods did not reach full thickness and the boundary between rod and interrod structure was not yet well defined **(C1,C1)**. In comparison, the structure of double-mutant enamel at the first and the second reference planes was similar to that observed at the second and the third reference planes, respectively, in the wild-type group **(A2,A2,B2,B2)**. There was a complete lack of decussating pattern in double-mutant enamel in the region labeled by the third reference plane, and aprismatic enamel/enamel-like structure dominated the whole vision field **(C2,C2)**.

For SEM analysis, we used the same reference planes as in the μCT analysis (Figures 2, 5). We exposed the surface of interest by fracturing the mandibular incisors in the coronal direction, which was consistent with the orientation of the reference planes. At the first and the second reference planes, wild-type enamel showed typical microstructure of maturation-stage enamel with rods and interrods laid out in a decussating and orderly pattern (Figures 5A1,A1',B1,B1'). In wild-type enamel at secretory stage, which was marked by the third reference plane, enamel rods did not reach full thickness and the boundary between rod and interrod structure was not yet well defined (Figures 5C1,C1). In comparison, the structure of double-mutant enamel at the first and the second reference planes was similar to that observed at the second and the third reference planes in wild-type group, respectively (Figures 5A2,A2',B2,B2'), suggesting the double knockout animals showed a delay in maturation. Furthermore, there was a complete lack of decussating pattern in double-mutant enamel in the region labeled by the third reference plane, and aprismatic enamel/enamel-like structure dominated the whole vision field (Figures 5C2,C2′).

### Double mutations impact mineral deposition

**Figure 6:**
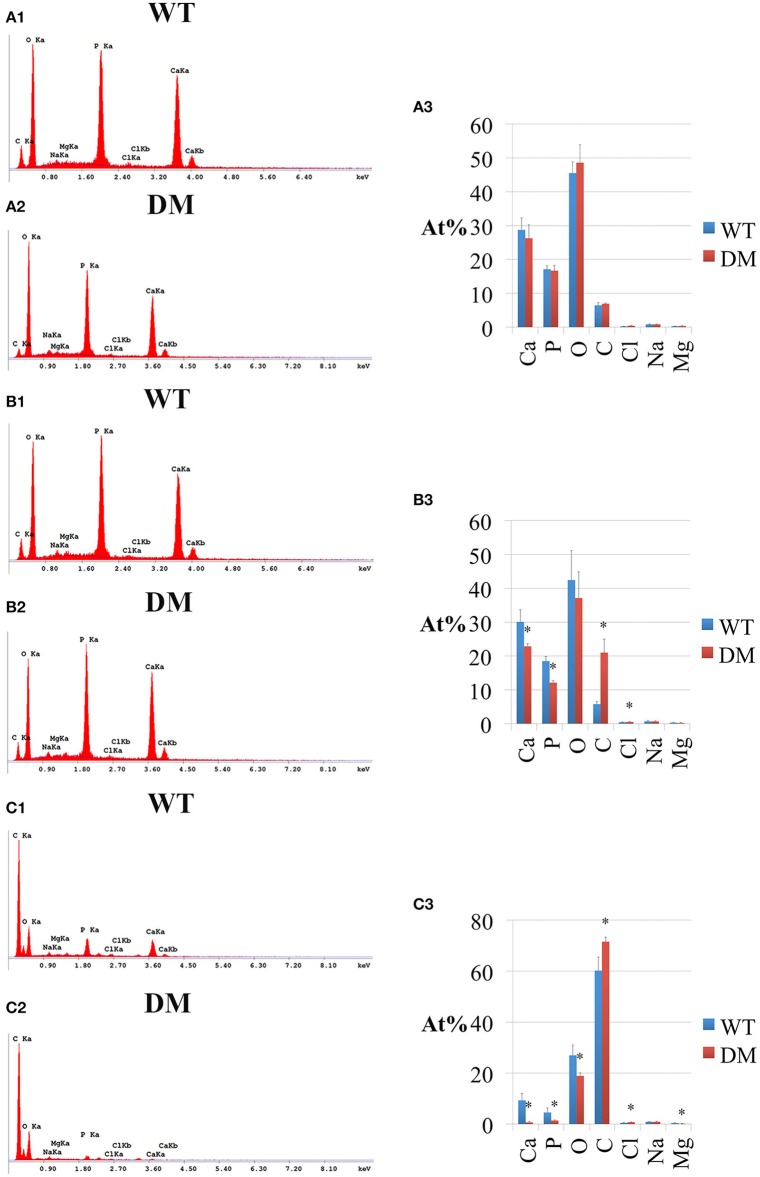
**EDS analysis of enamel in wild-type and double-mutant mandibular incisors**. WT, Wild-type; DM, Double-mutant; At%, Atomic percentage. **(A1,A2)** EDS spectrum of enamel at the first reference plane. **(A3)** Between wild-type and double-mutant enamel at the first reference plane, there were no statistically significant differences in the atomic percentages (At%) of all the elements analyzed—Ca, P, O, C, Cl, Na, and Mg. **(B1,B2)** EDS spectrum of enamel at the second reference plane. **(B3)** Statistically significant changes were detected in the At% of Ca, P, C, and Cl. The At% of Ca and P in double-mutant enamel decreased by ~26.7 and ~35.1%, respectively, compared to those in wild-type enamel. There were increases in the At% of C and Cl— ~268.4 and ~18.6%, respectively (double-mutant/wild-type). **(C1,C2)** EDS spectrum of enamel at the third reference plane. **(C3)** Double-mutant enamel showed significantly lower At% of Ca, P, O, Mg (~92.8, ~69.2, ~29.6, and ~57.9%), and higher At% of C and Cl (~20.0 and ~35.6%).

Following SEM, we analyzed the elemental compositions using EDS on the same regions of mandibular incisor enamel marked by the three previously mentioned reference planes (Figures 2, 5). At the first reference plane, there were no statistically significant differences between wild-type and double-mutant enamel in the atomic percentages (At%) of all the elements analyzed—Ca, P, O, C, Cl, Na, and Mg (Figures 6A1–A3). At the second reference plane, statistically significant changes were detected in the At% of Ca, P, C, and Cl (Figures 6B1–B3). The At% of Ca and P in double-mutant enamel decreased by ~26.7 and ~35.1%, respectively, compared to those in wild-type enamel (Figures 6B1–B3), while there were increases in the At% of C and Cl—~268.4 and ~18.6%, respectively (double-mutant/wild-type, Figures 6B1–B3). Changes in the elemental compositions at the third reference plane showed similar trends to those detected at the second reference plane—double-mutant enamel showed significantly lower At% of Ca, P, O, Mg (~92.8, ~69.2, ~29.6, and ~57.9%, Figures 6B1–C3), and higher At% of C and Cl (~20.0 and ~35.6%, Figures 6B1–C3).

### Deletion of *Slc26a1* and *Slc26a7* does not affect morphology of ameloblasts

**Figure 7:**
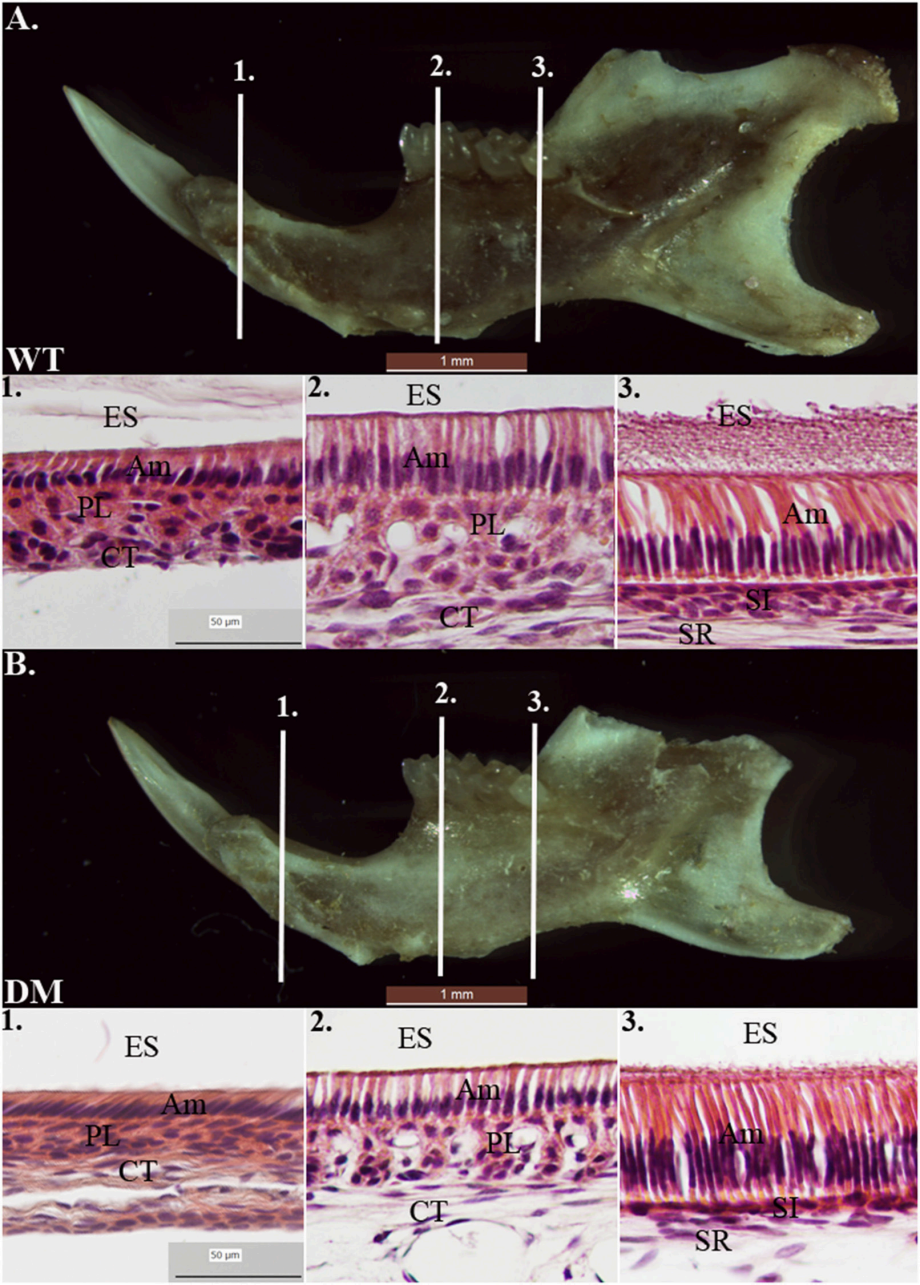
**Histological analysis of wild-type and double-mutant enamel organs by H& E staining**. WT, Wild-type; DM, Double-mutant; ES, Enamel space; Am, Ameloblast; PL, Papillary layer; CT, Connective tissue; SI, Stratum intermedium; SR, Stellate reticulum. **(A1–A3)** Wild-type enamel organ in late-maturation, maturation and secretory stages (labeled by three reference planes 1, 2, and 3). **(B1–B3)** Double-mutant enamel organ at the three reference planes. Magnification 40x. Compared to the wild-type group, cell morphology in double-mutant enamel organs in the same regions was not significantly different.

We prepared tissue sections from 4-week-old mouse mandibles (wild-type and double-mutant) for H & E staining. At the regions marked by the three reference planes, wild-type enamel organs demonstrated typical morphology of ameloblasts in late-maturation, maturation, and secretory stages (Figures 7A1–A3). Compared with the findings in the wild-type group, cell morphology in double-mutant enamel organs in the same regions was not significantly different (Figures 7B1–B3).

### pH regulators show compensatory expression in double-mutant animals

**Figure 8:**
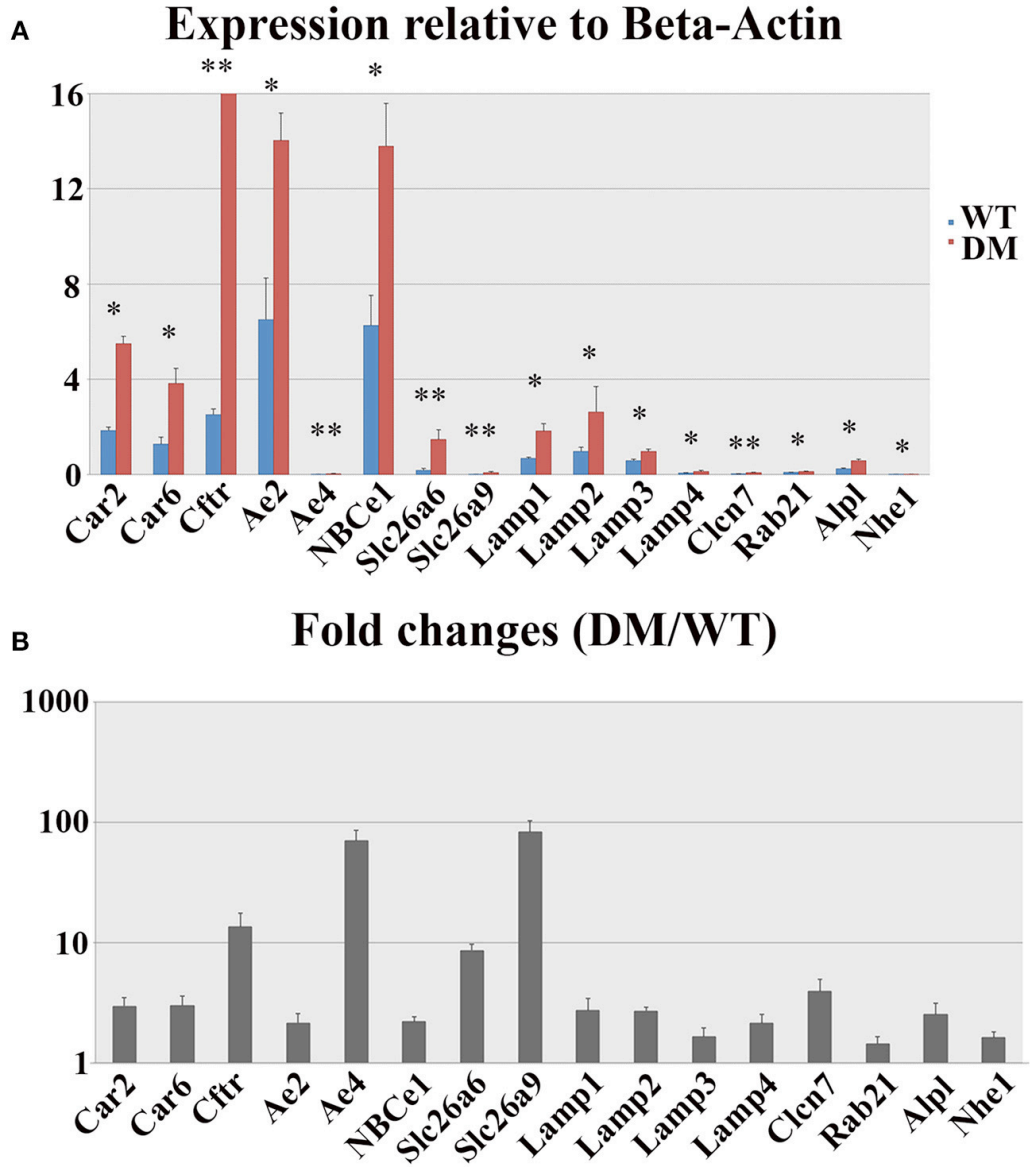
**Realtime PCR analysis of gene expression in wild-type and double-mutant maturation enamel organs**. WT, Wild-type; DM, Double-mutant. **(A)** Relative expression values normalized to that of *Beta-Actin*. **(B)** Fold changes. Significant upregulation was detected for all the genes quantified (DM/WT). *Ae4* and *Slc26a9* showed most striking fold changes—~70.5 and ~83.0%, respectively. The fold changes for the remaining genes were all above 2, except for *Lamp3, Rab21*, and *Nhe1*. ^*^P < 0.05; ^**^P < 0.01.

The expression levels of 16 genes involved in pH regulation and ion transport during maturation-stage amelogenesis were quantified by realtime PCR using RNA samples isolated from wild-type and double-mutant maturation-stage enamel organs. Significant upregulation was detected for all the genes quantified (double-mutant/wild-type, Figure 8). Note that *Ae4* and *Slc26a9* showed the most striking fold changes—~70.5 and ~83.0%, respectively (Figure 8B), and the fold changes for the remaining genes were all above 2, except for *Lamp3, Rab21*, and *Nhe1* (Figure 8B).

## Discussion

Enamel formation during maturation-stage amelogenesis involves mineral deposition, crystal growth, protease activities and the degradation of the internalized organic matrix, all of which are highly pH-dependent (Simmer and Fincham, 1995; Smith et al., 1996; Smith and Nanci, 1996; Lacruz et al., 2010a, 2012b). Acid-base balance in the extracellular matrix and intracellular lumens is maintained by a complex regulatory network involving multiple ion transporters and carbonic anhydrases (Dogterom and Bronckers, 1983; Lin et al., 1994; Wright et al., 1996a,b; Arquitt et al., 2002; Lyaruu et al., 2008; Paine et al., 2008; Bronckers et al., 2009, 2010; Josephsen et al., 2010; Wang et al., 2010; Lacruz et al., 2010a,b, 2012c, 2013a; Chang et al., 2011; Duan et al., 2011; Duan, 2014; Jalali et al., 2014; Reibring et al., 2014; Wen et al., 2014). Although details regarding the mechanism of pH control are yet to be clarified, the critical roles of many genes in maturation-stage pH regulation have been implicated by previous studies on transgenic animal models. For example, NBCe1 is a sodium-bicarbonate cotransporter expressed mainly on the basolateral membrane of maturation-stage ameloblasts (Lacruz et al., 2010b; Jalali et al., 2014). *NBCe1*^−/−^ animals demonstrated hypomineralized and weak enamel with an abnormal prismatic architecture. Severe enamel phenotypes have also been documented from *Cftr*^−/−^ and *Ae2*^−/−^ animals (Arquitt et al., 2002; Lyaruu et al., 2008; Bronckers et al., 2010; Chang et al., 2011). Bronckers et al. started to investigate the role of Slc26 family genes in tooth enamel formation in 2011 (Bronckers et al., 2011). Since then, all the animal studies on Slc26 mutations have reach similar conclusions: mutation or silencing of individual Slc26 gene members (*Slc26a1, Slc26a3, Slc26a4, Slc26a6*, and *Slc26a7*) is not sufficient to generate abnormal enamel phenotypes, yet the deletion of a single *Slc26* gene can induce strong compensatory expression of other pH regulatory genes and SLC26 family members (Bronckers et al., 2011; Jalali et al., 2015; Yin et al., 2015).

In the present study, we continued with our previous investigation of *Slc26a1*^−/−^ and *Slc26a7*^−/−^ animal models. We generated a double-null animal line with the absence of both *Slc26a1* and *Slc26a7* (*Slc26a1*^−/−^/*Slc26a7*^−/−^). The enamel density of double-null animals was significantly lower in the regions that represent late maturation-, maturation- and secretory-stage enamel development in age-matched wild-type siblings (Figures 2, 3). However, the difference in enamel density between double-mutant and wild-type mandibular first molars was not statistically significant, which suggests that incisor and molar maturation events differ to some extent (Figure 4). In addition, the “maturation” and “secretory” enamel microstructures in double-mutant animals resembled those observed in wild-type secretory and/or pre-secretory stages (Figure 5). This indicates that deletion of *Slc26a1* and S*lc26a7* delayed enamel development in mandibular incisors, although such an impact was not observed after full eruption of the mandibular first molars in double-mutant animals (only data from 4-week-old animals are shown in Figure 4). Subsequent elemental composition analysis of double-mutant incisors revealed that decreased enamel density could be attributed to a lack of mineral deposition (Ca^2+^ and HPO_3_, Figure 6). The accumulation of Cl^−^ in double-mutant enamel was consistent with our previous findings in *Slc26a1*^−/−^ and *Slc26a7*^−/−^ animals (Yin et al., 2015), while the increase of carbon in double-mutant enamel is a possible manifestation of disrupted EMP retrieval and hydrolysis (Figure 6). These findings indicate functional redundancy within the SLC26 gene family, and also in the scope of the pH regulatory network during enamel maturation (Yin et al., 2015). Such a redundancy is not uncommon in developmental processes and pathogenesis of diseases, e.g., matrix metalloproteinases (MMPs) in embryonic development and amyloid precursor protein (APP) genes in Alzheimer‘s disease (Heber et al., 2000; Page-McCaw et al., 2007).

Amelogenesis imperfecta (AI) is the most severe inherited disorder among all enamel pathologies. The genes responsible for AI in human patients include *AMELX, AMBN, ENAM, MMP20, KLK4, WDR72, FAM83H, LAMB3, ITGB6, and SLC24A4*, and current evidence tends to support a single-gene origin for many AI cases (Aldred et al., 1992; Lench et al., 1994; Lagerstrom-Fermer and Landegren, 1995; Lagerstrom-Fermer et al., 1995; Collier et al., 1997; MacDougall et al., 1997; Hart et al., 2000, 2003, 2004, 2009; Kindelan et al., 2000; Mardh et al., 2002; Kim et al., 2004, 2005, 2008, 2013; El-Sayed et al., 2009; Wright et al., 2009; Poulter et al., 2014a,b,c; Wang S. et al., 2014; Wang S. K. et al., 2014; Herzog et al., 2015). Our data from the double-mutant animals (*Slc26a1*^−/−^/*Slc26a7*^−/−^) suggest that polygenic etiologic factors might also be involved in the pathogenesis of AI/AI-like symptoms, which increases the complexity of genetic diagnosis for enamel disorders. The statement is further corroborated by the findings from a recent study on BMPs, in which double deletion of *Bmp2* and *Bmp4* in the epithelium led to hypoplastic enamel in mice (Xie et al., 2016).

We proposed in our previous study that pH regulation during enamel maturation might be achieved by the coordination of functional protein complexes (Yin et al., 2015). This is based on our findings that physical protein-protein interactions exist between Cftr and Slc26 gene family members *Slc26a1, Slc26a6*, and *Slc26a7* in maturation-stage ameloblasts, as supported by colocalization analyses, including co-immunofluorescence and co-immunoprecipitation studies. Here we examined the co-distribution pattern of SLC26A1 and SLC26A7 in maturation-stage ameloblasts by conducting immunostaining. We did not observe any overlaps in fluorescence of SLC26A1 and SLC26A7, which indicates a possible lack of colocalization of these two anion exchangers on the apical membrane of ameloblasts (Figure 1A). Nevertheless, the interactions between other different pH regulators in enamel maturation still warrants further investigation.

In conclusion, the data obtained from *Slc26a1*^−/−^/*Slc26a7*^−/−^ mutant mice provide new evidence in support of the hypothesis that SLC26A1 and SLC26A7 are actively involved in the ameloblast-mediated pH regulation process during maturation-stage amelogenesis.

## Author contributions

KY, JG, MS and MP designed the experiments; KY and WL performed the experiments; MS developed the mutant animal model; KY, JG, SR and MP analyzed the data; KY and JG prepared the Figures and tables; and KY and MP wrote the manuscript. All listed authors critically read, edited, and approved the final manuscript. MP accepts full responsibility for the integrity of the data analysis.

## Funding

This work was supported by NIH/NIDCR [grants # R01 DE019629 and R21 DE024704 (MP), R90 DE021982 (KY), and from the Department of Veterans Affairs [grant - Merit Review 5 I01 BX001000-06 award (MS)].

### Conflict of interest statement

The authors declare that the research was conducted in the absence of any commercial or financial relationships that could be construed as a potential conflict of interest.

## References

[B1] Aldred M. J. Crawford P. J. Roberts E. Gillespie C. M. Thomas N. S. Fenton I.. (1992). Genetic heterogeneity in X-linked amelogenesis imperfecta. Genomics 14, 567–573. 1358807 10.1016/s0888-7543(05)80153-3

[B2] Alper S. L. Sharma A. K. (2013). The SLC26 gene family of anion transporters and channels. Mol. Aspects Med. 34, 494–515. 10.1016/j.mam.2012.07.009 23506885 PMC3602804

[B3] Andrejewski N. Punnonen E. L. Guhde G. Tanaka Y. Lullmann-Rauch R. Hartmann D.. (1999). Normal lysosomal morphology and function in LAMP-1-deficient mice. J. Biol. Chem. 274, 12692–12701. 10212251 10.1074/jbc.274.18.12692

[B4] Arquitt C. K. Boyd C. Wright J. T. (2002). Cystic fibrosis transmembrane regulator gene (CFTR) is associated with abnormal enamel formation. J. Dent. Res. 81, 492–496. 10.1177/154405910208100712 12161463

[B5] Bertrand C. A. Zhang R. Pilewski J. M. Frizzell R. A. (2009). SLC26A9 is a constitutively active, CFTR-regulated anion conductance in human bronchial epithelia. J. Gen. Physiol. 133, 421–438. 10.1085/jgp.200810097 19289574 PMC2664976

[B6] Bronckers A. Kalogeraki L. Jorna H. J. Wilke M. Bervoets T. J. Lyaruu D. M.. (2010). The cystic fibrosis transmembrane conductance regulator (CFTR) is expressed in maturation stage ameloblasts, odontoblasts and bone cells. Bone 46, 1188–1196. 10.1016/j.bone.2009.12.002 20004757 PMC2842452

[B7] Bronckers A. L. Guo J. Zandieh-Doulabi B. Bervoets T. J. Lyaruu D. M. Li X.. (2011). Developmental expression of solute carrier family 26A member 4 (SLC26A4/pendrin) during amelogenesis in developing rodent teeth. Eur. J. Oral. Sci. 119 (Suppl. 1), 185–192. 10.1111/j.1600-0722.2011.00901.x 22243245 PMC3496853

[B8] Bronckers A. L. Lyaruu D. M. Jansen I. D. Medina J. F. Kellokumpu S. Hoeben K. A.. (2009). Localization and function of the anion exchanger Ae2 in developing teeth and orofacial bone in rodents. J. Exp. Zool. B Mol. Dev. Evol. 312B, 375–387. 10.1002/jez.b.21267 19206174 PMC3142622

[B9] Chang E. H. Lacruz R. S. Bromage T. G. Bringas P. Jr. Welsh M. J. Zabner J.. (2011). Enamel pathology resulting from loss of function in the cystic fibrosis transmembrane conductance regulator in a porcine animal model. Cells Tiss. Organs. 194, 249–254. 10.1159/000324248 21525720 PMC3178086

[B10] Collier P. M. Sauk J. J. Rosenbloom S. J. Yuan Z. A. Gibson C. W. (1997). An amelogenin gene defect associated with human X-linked amelogenesis imperfecta. Arch. Oral Biol. 42, 235–242. 9188994 10.1016/s0003-9969(96)00099-4

[B11] Dawson P. A. Russell C. S. Lee S. McLeay S. C. van Dongen J. M. Cowley D. M.. (2010). Urolithiasis and hepatotoxicity are linked to the anion transporter Sat1 in mice. J. Clin. Invest. 120, 706–712. 10.1172/JCI31474 20160351 PMC2827940

[B12] Dogterom A. A. Bronckers A. L. (1983). Carbonic anhydrase in developing hamster molars. J. Dent. Res. 62, 789–791. 6408149 10.1177/00220345830620070201

[B13] Duan X. (2014). Ion channels, channelopathies, and tooth formation. J. Dent. Res. 93, 117–125. 10.1177/0022034513507066 24076519

[B14] Duan X. Mao Y. Wen X. Yang T. Xue Y. (2011). Excess fluoride interferes with chloride-channel-dependent endocytosis in ameloblasts. J. Dent. Res. 90, 175–180. 10.1177/0022034510385687 21148016

[B15] El-Sayed W. Parry D. A. Shore R. C. Ahmed M. Jafri H. Rashid Y.. (2009). Mutations in the beta propeller WDR72 cause autosomal-recessive hypomaturation amelogenesis imperfecta. Am. J. Hum. Genet. 85, 699–705. 10.1016/j.ajhg.2009.09.014 19853237 PMC2775821

[B16] Freel R. W. Hatch M. Green M. Soleimani M. (2006). Ileal oxalate absorption and urinary oxalate excretion are enhanced in Slc26a6 null mice. Am. J. Physiol. Gastrointest. Liver Physiol. 290, G719–728. 10.1152/ajpgi.00481.2005 16373425

[B17] Hart P. S. Becerik S. Cogulu D. Emingil G. Ozdemir-Ozenen D. Han S. T.. (2009). Novel FAM83H mutations in Turkish families with autosomal dominant hypocalcified amelogenesis imperfecta. Clin. Genet. 75, 401–404. 10.1111/j.1399-0004.2008.01112.x 19220331 PMC4264522

[B18] Hart P. S. Hart T. C. Michalec M. D. Ryu O. H. Simmons D. Hong S.. (2004). Mutation in kallikrein 4 causes autosomal recessive hypomaturation amelogenesis imperfecta. J. Med. Genet. 41, 545–549. 10.1136/jmg.2003.017657 15235027 PMC1735847

[B19] Hart S. Hart T. Gibson C. Wright J. T. (2000). Mutational analysis of X-linked amelogenesis imperfecta in multiple families. Arch. Oral Biol. 45, 79–86. 10.1016/S0003-9969(99)00106-5 10669095

[B20] Hart T. C. Hart P. S. Gorry M. C. Michalec M. D. Ryu O. H. Uygur C.. (2003). Novel ENAM mutation responsible for autosomal recessive amelogenesis imperfecta and localised enamel defects. J. Med. Genet. 40, 900–906. 10.1136/jmg.40.12.900 14684688 PMC1735344

[B21] Heber S. Herms J. Gajic V. Hainfellner J. Aguzzi A. Rulicke T. (2000). Mice with combined gene knock-outs reveal essential and partially redundant functions of amyloid precursor protein family members. J. Neurosci. 20, 7951–7963. 11050115 10.1523/JNEUROSCI.20-21-07951.2000 PMC6772747

[B22] Herzog C. R. Reid B. M. Seymen F. Koruyucu M. Tuna E. B. Simmer J. P.. (2015). Hypomaturation amelogenesis imperfecta caused by a novel SLC24A4 mutation. Oral Surg. Oral Med. Oral Pathol. Oral Radiol. 119, e77–e81. 10.1016/j.oooo.2014.09.003 25442250 PMC4291293

[B23] Jalali R. Guo J. Zandieh-Doulabi B. Bervoets T. J. Paine M. L. Boron W. F.. (2014). NBCe1 (SLC4A4) a potential pH regulator in enamel organ cells during enamel development in the mouse. Cell Tissue Res. 358, 433–442. 10.1007/s00441-014-1935-4 25012520 PMC5142611

[B24] Jalali R. Zandieh-Doulabi B. DenBesten P. K. Seidler U. Riederer B. Wedenoja S.. (2015). Slc26a3/Dra and Slc26a6 in Murine Ameloblasts. J. Dent. Res. 94, 1732–1739. 10.1177/0022034515606873 26394631 PMC4681479

[B25] Jiang Z. Asplin J. R. Evan A. P. Rajendran V. M. Velazquez H. Nottoli T. P.. (2006). Calcium oxalate urolithiasis in mice lacking anion transporter Slc26a6. Nat. Genet. 38, 474–478. 10.1038/ng1762 16532010

[B26] Josephsen K. Takano Y. Frische S. Praetorius J. Nielsen S. Aoba T.. (2010). Ion transporters in secretory and cyclically modulating ameloblasts: a new hypothesis for cellular control of preeruptive enamel maturation. Am. J. Physiol., Cell Physiol. 299, C1299–1307. 10.1152/ajpcell.00218.2010 20844245

[B27] Kim J. W. Lee S. K. Lee Z. H. Park J. C. Lee K. E. Lee M. H.. (2008). FAM83H mutations in families with autosomal-dominant hypocalcified amelogenesis imperfecta. Am. J. Hum. Genet. 82, 489–494. 10.1016/j.ajhg.2007.09.020 18252228 PMC2427219

[B28] Kim J. W. Seymen F. Lee K. E. Ko J. Yildirim M. Tuna E. B.. (2013). LAMB3 mutations causing autosomal-dominant amelogenesis imperfecta. J. Dent. Res. 92, 899–904. 10.1177/0022034513502054 23958762 PMC3775375

[B29] Kim J. W. Simmer J. P. Hart T. C. Hart P. S. Ramaswami M. D. Bartlett J. D.. (2005). MMP-20 mutation in autosomal recessive pigmented hypomaturation amelogenesis imperfecta. J. Med. Genet. 42, 271–275. 10.1136/jmg.2004.024505 15744043 PMC1736010

[B30] Kim J. W. Simmer J. P. Hu Y. Y. Lin B. P. Boyd C. Wright J. T. (2004). Amelogenin p.M1T and p.W4S mutations underlying hypoplastic X-linked amelogenesis imperfecta. J. Dent. Res. 83, 378–383. 10.1177/154405910408300505 15111628

[B31] Kindelan S. A. Brook A. H. Gangemi L. Lench N. Wong F. S. Fearne J.. (2000). Detection of a novel mutation in X-linked amelogenesis imperfecta. J. Dent. Res. 79, 1978–1982. 10.1177/00220345000790120901 11201048

[B32] Lacruz R. S. Brookes S. J. Wen X. Jimenez J. M. Vikman S. Hu P.. (2013a). Adaptor protein complex 2-mediated, clathrin-dependent endocytosis, and related gene activities, are a prominent feature during maturation stage amelogenesis. J. Bone Miner. Res. 28, 672–687. 10.1002/jbmr.1779 23044750 PMC3562759

[B33] Lacruz R. S. Nakayama Y. Holcroft J. Nguyen V. Somogyi-Ganss E. Snead M. L.. (2012a). Targeted overexpression of amelotin disrupts the microstructure of dental enamel. PLoS ONE 7:e35200. 10.1371/journal.pone.0035200 22539960 PMC3335167

[B34] Lacruz R. S. Nanci A. Kurtz I. Wright J. T. Paine M. L. (2010a). Regulation of pH During Amelogenesis. Calcif. Tiss. Int. 86, 91–103. 10.1007/s00223-009-9326-7 20016979 PMC2809306

[B35] Lacruz R. S. Nanci A. White S. N. Wen X. Wang H. Zalzal S. F.. (2010b). The sodium bicarbonate cotransporter (NBCe1) is essential for normal development of mouse dentition. J. Biol. Chem. 285, 24432–24438. 10.1074/jbc.M110.115188 20529845 PMC2915679

[B36] Lacruz R. S. Smith C. E. Bringas P. Jr. Chen Y. B. Smith S. M. Snead M. L.. (2012b). Identification of novel candidate genes involved in mineralization of dental enamel by genome-wide transcript profiling. J. Cell. Physiol. 227, 2264–2275. 10.1002/jcp.22965 21809343 PMC3243804

[B37] Lacruz R. S. Smith C. E. Chen Y. B. Hubbard M. J. Hacia J. G. Paine M. L. (2011). Gene-expression analysis of early- and late-maturation-stage rat enamel organ. Eur. J. Oral. Sci. 119 (Suppl. 1), 149–157. 10.1111/j.1600-0722.2011.00881.x 22243241 PMC3286129

[B38] Lacruz R. S. Smith C. E. Kurtz I. Hubbard M. J. Paine M. L. (2013b). New paradigms on the transport functions of maturation-stage ameloblasts. J. Dent. Res. 92, 122–129. 10.1177/0022034512470954 23242231 PMC3545694

[B39] Lacruz R. S. Smith C. E. Moffatt P. Chang E. H. Bromage T. G. Bringas P. Jr.. (2012c). Requirements for ion and solute transport, and pH regulation during enamel maturation. J. Cell. Physiol. 227, 1776–1785. 10.1002/jcp.22911 21732355 PMC3373187

[B40] Lagerstrom-Fermer M. Landegren U. (1995). Understanding enamel formation from mutations causing X-linked amelogenesis imperfecta. Connect. Tissue Res. 32, 241–246. 7554922 10.3109/03008209509013729

[B41] Lagerstrom-Fermer M. Nilsson M. Backman B. Salido E. Shapiro L. Pettersson U.. (1995). Amelogenin signal peptide mutation: correlation between mutations in the amelogenin gene (AMGX) and manifestations of X-linked amelogenesis imperfecta. Genomics 26, 159–162. 7782077 10.1016/0888-7543(95)80097-6

[B42] Lench N. J. Brook A. H. Winter G. B. (1994). SSCP detection of a nonsense mutation in exon 5 of the amelogenin gene (AMGX) causing X-linked amelogenesis imperfecta (AIH1). Hum. Mol. Genet. 3, 827–828. 8081371 10.1093/hmg/3.5.827

[B43] Lezot F. Thomas B. Greene S. R. Hotton D. Yuan Z. A. Castaneda B.. (2008). Physiological implications of DLX homeoproteins in enamel formation. J. Cell. Physiol. 216, 688–697. 10.1002/jcp.21448 18366088

[B44] Lin H. M. Nakamura H. Noda T. Ozawa H. (1994). Localization of H(+)-ATPase and carbonic anhydrase II in ameloblasts at maturation. Calcif. Tissue Int. 55, 38–45. 7922788 10.1007/BF00310167

[B45] Livak K. J. Schmittgen T. D. (2001). Analysis of relative gene expression data using real-time quantitative PCR and the 2(-Delta Delta C(T)) Method. Methods 25, 402–408. 10.1006/meth.2001.1262 11846609

[B46] Lloyd J. B. (1996). Metabolite efflux and influx across the lysosome membrane. Subcell. Biochem. 27, 361–386. 8993166 10.1007/978-1-4615-5833-0_11

[B47] Lyaruu D. M. Bronckers A. L. Mulder L. Mardones P. Medina J. F. Kellokumpu S.. (2008). The anion exchanger Ae2 is required for enamel maturation in mouse teeth. Matrix Biol. 27, 119–127. 10.1016/j.matbio.2007.09.006 18042363 PMC2274829

[B48] MacDougall M. DuPont B. R. Simmons D. Reus B. Krebsbach P. Karrman C.. (1997). Ameloblastin gene (AMBN) maps within the critical region for autosomal dominant amelogenesis imperfecta at chromosome 4q21. Genomics 41, 115–118. 10.1006/geno.1997.4643 9126491

[B49] Mardh C. K. Backman B. Holmgren G. Hu J. C. Simmer J. P. Forsman-Semb K. (2002). A nonsense mutation in the enamelin gene causes local hypoplastic autosomal dominant amelogenesis imperfecta (AIH2). Hum. Mol. Genet. 11, 1069–1074. 10.1093/hmg/11.9.1069 11978766

[B50] Nanci A. (2008). Ten Cate's oral Histology Development, Structure and Function. St Louis, MO: Mosby Elsevier.

[B51] Page-McCaw A. Ewald A. J. Werb Z. (2007). Matrix metalloproteinases and the regulation of tissue remodelling. Nat. Rev. Mol. Cell Biol. 8, 221–233. 10.1038/nrm2125 17318226 PMC2760082

[B52] Paine M. L. Snead M. L. Wang H. J. Abuladze N. Pushkin A. Liu W.. (2008). Role of NBCe1 and AE2 in secretory ameloblasts. J. Dent. Res. 87, 391–395. 10.1177/154405910808700415 18362326 PMC2504252

[B53] Petrovic S. Barone S. Xu J. Conforti L. Ma L. Kujala M.. (2004). SLC26A7: a basolateral Cl-/HCO3- exchanger specific to intercalated cells of the outer medullary collecting duct. Am. J. Physiol. Renal Physiol. 286, F161–169. 10.1152/ajprenal.00219.2003 12965893

[B54] Petrovic S. Ju X. Barone S. Seidler U. Alper S. L. Lohi H.. (2003a). Identification of a basolateral Cl-/HCO3- exchanger specific to gastric parietal cells. Am. J. Physiol. Gastrointest. Liver Physiol. 284, G1093–1103. 10.1152/ajpgi.00454.2002 12736153

[B55] Petrovic S. Ma L. Wang Z. Soleimani M. (2003b). Identification of an apical Cl-/HCO-3 exchanger in rat kidney proximal tubule. Am. J. Physiol. Cell Physiol. 285, C608–617. 10.1152/ajpcell.00084.2003 12736136

[B56] Poulter J. A. Brookes S. J. Shore R. C. Smith C. E. Abi Farraj L. Kirkham J.. (2014a). A missense mutation in ITGB6 causes pitted hypomineralized amelogenesis imperfecta. Hum. Mol. Genet. 23, 2189–2197. 10.1093/hmg/ddt616 24319098 PMC3959822

[B57] Poulter J. A. El-Sayed W. Shore R. C. Kirkham J. Inglehearn C. F. Mighell A. J. (2014b). Whole-exome sequencing, without prior linkage, identifies a mutation in LAMB3 as a cause of dominant hypoplastic amelogenesis imperfecta. Eur. J. Hum. Genet. 22, 132–135. 10.1038/ejhg.2013.76 23632796 PMC3865405

[B58] Poulter J. A. Murillo G. Brookes S. J. Smith C. E. Parry D. A. Silva S. (2014c). Deletion of ameloblastin exon 6 is associated with amelogenesis imperfecta. Hum. Mol. Genet. 23, 5317–5324. 10.1093/hmg/ddu247 24858907 PMC4168819

[B59] Reibring C. G. El Shahawy M. Hallberg K. Kannius-Janson M. Nilsson J. Parkkila S.. (2014). Expression patterns and subcellular localization of carbonic anhydrases are developmentally regulated during tooth formation. PLoS ONE 9:e96007. 10.1371/journal.pone.0096007 24789143 PMC4006843

[B60] Schmittgen T. D. Livak K. J. (2008). Analyzing real-time PCR data by the comparative C(T) method. Nat. Protoc. 3, 1101–1108. 10.1038/nprot.2008.73 18546601

[B61] Simmer J. P. Fincham A. G. (1995). Molecular mechanisms of dental enamel formation. Crit. Rev. Oral Biol. Med. 6, 84–108. 7548623 10.1177/10454411950060020701

[B62] Smith C. E. Issid M. Margolis H. C. Moreno E. C. (1996). Developmental changes in the pH of enamel fluid and its effects on matrix-resident proteinases. Adv. Dent. Res. 10, 159–169. 9206332 10.1177/08959374960100020701

[B63] Smith C. E. Nanci A. (1996). Protein dynamics of amelogenesis. Anat. Rec. 245, 186–207. 10.1002/(SICI)1097-0185(199606)245:2<186::AID-AR7>3.0.CO;2-V 8769663

[B64] Wang S. Choi M. Richardson A. S. Reid B. M. Seymen F. Yildirim M.. (2014). STIM1 and SLC24A4 are critical for enamel maturation. J. Dent. Res. 93(7 Suppl.), 94S–100S. 10.1177/0022034514527971 24621671 PMC4107542

[B65] Wang S. K. Choi M. Richardson A. S. Reid B. M. Lin B. P. Wang S. J.. (2014). ITGB6 loss-of-function mutations cause autosomal recessive amelogenesis imperfecta. Hum. Mol. Genet. 23, 2157–2163. 10.1093/hmg/ddt611 24305999 PMC3959820

[B66] Wang X. Suzawa T. Ohtsuka H. Zhao B. Miyamoto Y. Miyauchi T.. (2010). Carbonic anhydrase II regulates differentiation of ameloblasts via intracellular pH-dependent JNK signaling pathway. J. Cell. Physiol. 225, 709–719. 10.1002/jcp.22267 20533306

[B67] Wen X. Kurtz I. Paine M. L. (2014). Prevention of the disrupted enamel phenotype in Slc4a4-null mice using explant organ culture maintained in a living host kidney capsule. PLoS ONE 9:e97318. 10.1371/journal.pone.0097318 24828138 PMC4020772

[B68] Wen X. Lacruz R. S. Paine M. L. (2015). Dental and Cranial Pathologies in Mice Lacking the Cl^−^ /H^+^ -Exchanger ClC-7. Anat. Rec. (Hoboken). 298, 1502–1508. 10.1002/ar.23118 25663454 PMC4503507

[B69] Wright J. T. Frazier-Bowers S. Simmons D. Alexander K. Crawford P. Han S. T.. (2009). Phenotypic variation in FAM83H-associated amelogenesis imperfecta. J. Dent. Res. 88, 356–360. 10.1177/0022034509333822 19407157 PMC2754853

[B70] Wright J. T. Hall K. I. Grubb B. R. (1996a). Enamel mineral composition of normal and cystic fibrosis transgenic mice. Adv. Dent. Res. 10, 270–274; discussion 275. 9206347 10.1177/08959374960100022501

[B71] Wright J. T. Kiefer C. L. Hall K. I. Grubb B. R. (1996b). Abnormal enamel development in a cystic fibrosis transgenic mouse model. J. Dent. Res. 75, 966–973. 8708137 10.1177/00220345960750041101

[B72] Xie Q. Welch R. Mercado A. Romero M. F. Mount D. B. (2002). Molecular characterization of the murine Slc26a6 anion exchanger: functional comparison with Slc26a1. Am. J. Physiol. Renal Physiol. 283, F826–F838. 10.1152/ajprenal.00079.2002 12217875

[B73] Xie X. Liu C. Zhang H. Jani P. H. Lu Y. Wang X.. (2016). Abrogation of epithelial BMP2 and BMP4 causes Amelogenesis Imperfecta by reducing MMP20 and KLK4 expression. Sci. Rep. 6:25364. 10.1038/srep25364 27146352 PMC4857113

[B74] Xu J. Song P. Nakamura S. Miller M. Barone S. Alper S. L. (2009). Deletion of the chloride transporter slc26a7 causes distal renal tubular acidosis and impairs gastric acid secretion. J. Biol. Chem. 284, 29470–29479. 10.1074/jbc.M109.044396 19723628 PMC2785580

[B75] Yin K. Hacia J. G. Zhong Z. Paine M. L. (2014). Genome-wide analysis of miRNA and mRNA transcriptomes during amelogenesis. BMC Genomics 15:998. 10.1186/1471-2164-15-998 25406666 PMC4254193

[B76] Yin K. Lei Y. Wen X. Lacruz R. S. Soleimani M. Kurtz I.. (2015). SLC26A Gene family participate in pH regulation during enamel maturation. PLoS ONE 10:e0144703. 10.1371/journal.pone.0144703 26671068 PMC4679777

